# Role of the ubiquitin proteasome system in Alzheimer's disease

**DOI:** 10.1186/1471-2091-8-S1-S12

**Published:** 2007-11-22

**Authors:** Sudarshan C Upadhya, Ashok N Hegde

**Affiliations:** 1 Department of Neurobiology and Anatomy, Wake Forest University Health Sciences Medical Center Boulevard, Winston-Salem, NC 27157, USA

## Abstract

Though Alzheimer's disease (AD) is a syndrome with well-defined clinical and neuropathological manifestations, an array of molecular defects underlies its pathology. A role for the ubiquitin proteasome system (UPS) was suspected in the pathogenesis of AD since the presence of ubiquitin immunoreactivity in AD-related neuronal inclusions, such as neurofibrillary tangles, is seen in all AD cases. Recent studies have indicated that components of the UPS could be linked to the early phase of AD, which is marked by synaptic dysfunction, as well as to the late stages of the disease, characterized by neurodegeneration. Insoluble protein aggregates in the brain of AD patients could result from malfunction or overload of the UPS, or from structural changes in the protein substrates, which prevent their recognition and degradation by the UPS. Defective proteolysis could cause the synaptic dysfunction observed early in AD since the UPS is known to play a role in the normal functioning of synapses. In this review, we discuss recent observations on possible links between the UPS and AD, and the potential for utilizing UPS components as targets for treatment of this disease.

**Publication history:** Republished from Current BioData's Targeted Proteins database (TPdb; ).

## Protein pathway in the disease - Introduction

The ubiquitin proteasome system (UPS) plays a role in a variety of cellular functions. In the UPS, substrate proteins are targeted for degradation by covalent attachment of ubiquitin, which is mediated by an enzymatic cascade consisting of activating (E1), conjugating (E2) and ligating (E3) enzymes. The ubiquitin-conjugated proteins are subsequently degraded by a large multi-subunit complex, the 26S proteasome. Substrate-specific E3s, along with specific E2s, ensure selective protein targeting for proteolysis [1,2]. In the nervous system, the UPS plays a role in normal physiological function, while evidence gathered in the past several years also indicates a role in neurodegenerative diseases such as Alzheimer's disease (AD) [1,2]. In this article, we discuss AD pathogenesis and how the UPS could be linked to the early and late stages of the disease. We also suggest how further research into this area might help to develop therapeutic strategies for AD.

## Alzheimer's disease

AD is a neurodegenerative disorder of the CNS, clinically characterized by progressive loss of memory and other cognitive skills, resulting in severe dementia. The condition often begins with mild memory lapses and then gradually advances to dementia: a progressive deterioration of memory, language and most mental functions. During the early stages of AD development, neurological examination is normal except for observed cognitive dysfunctions such as progressive worsening of memory [3-8]. The intellectual decline is accompanied by the progressive extracellular and intracellular accumulation of insoluble fibrous material in the brain in the form of senile plaques and neurofibrillary tangles (NFTs) [7].

**Figure 1:**
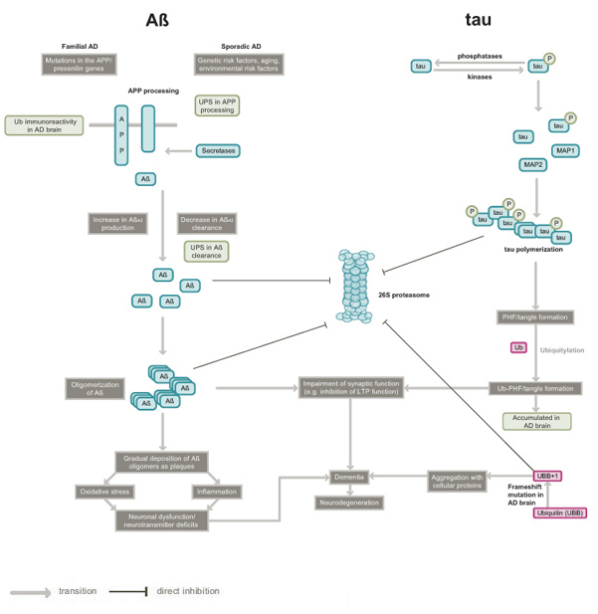
**Pathogenesis of Alzheimer's disease: potential roles of the ubiquitin proteasome system**. The figure summarizes the two major hypotheses (Aβ and tau) of AD pathology and the linkage of UPS to AD pathogenesis. Extracellular amyloid plaques consisting of insoluble Aβ peptide and intracellular neurofibrillary tangles comprising hyperphosphorylated protein tau are the two major features evident in the post mortem AD brain. Although the figure depicts only increased production of Aβ_42_ (a splice variant of Aβ, some familial Alzheimer's disease (FAD) mutations in APP or PSEN 1 also lead to increased Aβ_42_ secretion [71]. The roles of the UPS in the steps leading to AD pathogenesis are shown in green boxes. The ubiquitin mutant UBB^+1^ is also linked to AD, though it is unclear at present how pathogenesis mediated by UBB^+1^ relates to the major AD hypotheses. Abbreviations used are: Aβ, amyloid β; AD, Alzheimer's disease; APP, amyloid precursor protein; MAP, microtubule associated protein; PHFs, paired helical filaments; Ub, ubiquitin; UPS, ubiquitin proteasome system.

AD is the most prevalent neurodegenerative disorder and the most common cause of dementia [8]. Familial AD is a rare autosomal dominant disease with early onset, caused by mutations in the amyloid precursor protein (APP) and presenilin (PSEN) genes, both of which are linked to amyloid β (Aβ) peptide metabolism [9]. Aβ is produced from APP by sequential cleavage involving β-secretase (also known as β-site APP cleaving enzyme 1 (BACE1)) and γ-secretase (presenilin complex), and released into the extracellular spaces [10]. Sporadic AD is a common heterogeneous disease and is caused by a complex interaction of genetic and environmental risk factors [9] (see figure 1).

The pathological signs of both types of AD include the loss of medium and large pyramidal neurons, the presence of plaques and NFTs (composed of deposits of amyloid filaments and hyperphosphorylated tau, respectively, surrounded by altered neurite processes and glia), a degeneration of the neurons and the loss of synapses [11,12]. Tau is a structural protein that is normally associated with microtubuli. In atypical conditions (such as in AD), tau protein synthesis is upregulated and it undergoes an abnormal post-translational modification characterized by hyperphosphorylation. Although multiple genetic disturbances are believed to underlie AD, a major cause of the disease is buildup of the toxic Aβ peptide [13].

The formation of the neurofibrillary lesions is believed to lead to the symptoms of the disease, which result most probably from the degeneration of nerve cells in the cerebral cortex and hippocampal formation, following synaptic loss. Defective synaptic function and memory loss is seen in the early stages of human AD and in animal models of the disease; as such, cognitive deficits are correlated with the loss of synapses [14]. During the early phase of AD there is a 25–35% decrease in the density of synapses and as the disease progresses synaptic loss is more strongly correlated with the disease than the plaques and tangles [15]. Animal models of AD mimic the cognitive impairment seen in human AD [16,17].

Recent studies also suggest that AD begins with subtle alterations in synaptic efficacy prior to neuronal degeneration and that diffusible oligomeric assemblies of Aβ [14] are the cause of this neuronal dysfunction. Although there is eventual synaptic loss in both human AD and animal models of the disease, deficits in spatial memory and inhibition of long-term potentiation (LTP; an electrophysiological measure of synaptic strength) precede morphological alterations in the animal models, suggesting earlier biochemical changes in the disease [14,18,19]. Aβ treatment of cultured hippocampal neurons leads to the inactivation of protein kinase A (PKA). Persistence of its regulatory subunit PKAII alpha suggests that Aβ acts directly on the signaling pathways consisting of cAMP/PKA/cAMP-responsive element binding protein (CREB), which are required for development of late phase LTP [20]. Therefore, agents that enhance the PKA pathway have potential for the treatment of AD [20]. In transgenic mice expressing mutant APP, learning and memory is impaired at 9–10 months of age (although no Aβ plaques are observed in the brains of these mice) [21]. In mice carrying various mutations of APP, both *in vitro* and *in vivo* LTP is impaired much before detectable Aβ deposits are observed [22]. Also, cerebral microinjection of oligomers of Aβ peptide inhibits *in vivo* LTP in rats [23].

## The UPS and Alzheimer's disease

Several studies implicate neuronal UPS in the pathogenesis of AD (see figure 1). Abnormal deposition of highly insoluble protein aggregates or inclusion bodies within nerve cells is commonly observed in nervous tissue in association with several chronic neurodegenerative diseases including AD [1]. These inclusions bodies show ubiquitin immunoreactivity in human AD brains, along with immunoreactivity to other proteins [24-27]. The accumulation of ubiquitin–protein conjugates in neuropathological lesions was first detected in NFTs isolated from human brain [28,29], and it is these NFTs that best correlate with the degree of dementia [30]. It has been shown that aggregated proteins in cell line experiments tend to inhibit the UPS [31,32]. Alterations in the UPS have been connected to several neurodegenerative diseases [2,33]; in some instances, mutations in specific genes have been linked to the etiology of the disease while in other cases impairment of the UPS could be a late event in pathogenesis [32]. Although the perturbations in UPS-mediated proteolysis lead to pleiotropic effects on neurons including cell death or degeneration, one of the early effects is believed to be synaptic malfunction [14]. Many recent reports from animal studies demonstrate a role for UPS-mediated degradation of numerous substrate proteins in synaptic plasticity, thus linking the UPS to normal synaptic function [2,34]. For example, a *Drosophila* study suggested that the UPS acutely regulates presynaptic protein turnover and synaptic efficacy [35]. The UPS can also regulate long-term synaptic plasticity by degrading a crucial CREB repressor in *Aplysia,* as shown by investigations in our laboratory [36].

## Disease models, knockouts, assays

### The UPS and synaptic dysfunction in early Alzheimer's disease

Memory impairment in AD likely results from synaptic dysfunction, for example a defect in the ability to change synaptic strength or synaptic plasticity. The role of the UPS in synaptic plasticity was first observed in an invertebrate cellular model of long-term memory called long-term facilitation (LTF) [37]. Specifically, the regulatory subunits of PKA were found to be substrates for the UPS [37]. Also, *Aplysia* ubiquitin C-terminal hydrolase (Ap-uch), a homolog of vertebrate UCHL1 (ubiquitin carboxyl-terminal hydrolase isozyme L1), was found to enhance the degradation of polyubiquitylated R subunit of PKA by removing ubiquitin prior to degradation [38]. Direct electrophysiological experiments in which expression or function of Ap-Uch was perturbed demonstrated a crucial role for the hydrolase in LTF [38]. These studies have been corroborated by experiments on long-term memory in mice. Furthermore, synaptic dysfunction in hippocampal slices from APP/PSEN1 mice (an animal model of AD), or synaptic dysfunction in hippocampal slices from normal mice caused by treatment with oligomeric Aβ, was reversed by treatment with exogenous UCHL1 [39]. The basic research on the function of UCHL1 in synaptic plasticity [38], and the application of this observation to treat synaptic dysfunction in AD [39], suggests that perturbations in UPS components could be an important causative factor in AD.

A role for UCHL1 in AD has been supported by other observations. For example, the genetic defect in a mouse model of gracile axonal dystrophy (gad) was shown to be caused by an in-frame deletion that includes exons 7 and 8 of *Uchl1*, encoding UCHL1. The gad mice display ataxia (lack of coordination in muscular movements), characterized by dragging of the hind legs. In the sensory and motor neurons of gad mice, accumulation of Aβ and ubiquitin-positive deposits occurs in a manner reminiscent of AD pathology [40,41]. The studies by Osaka *et al.*[42] demonstrated that UCHL1 ensures ubiquitin stability within neurons. It was also shown that UCHL1 protein level is down-regulated in idiopathic Parkinson's disease (PD) as well as in AD brains [43]. Full-length UCHL1 appears to be a major target of oxidative damage in AD and PD brains, and the enzyme is extensively modified by carbonyl formation, methionine oxidation and cysteine oxidation [44]. Also, evidence from studies on Chinese Han population indicates a genetic association between a serine to tyrosine mutation (S18Y polymorphism) in *UCHL1* and sporadic AD. This study concluded that the Y allele and YY genotype of the S18Y polymorphism in *UCHL1* could confer a protective effect against sporadic AD in female subjects [45].

### The UPS and proteolytic defects in Alzheimer's disease

The ubiquitylated protein aggregates found in AD brains are likely to result from a malfunction or overload of the UPS or from structural changes in the protein substrates, halting their degradation. The presence of ubiquitylated proteins in all the AD cases [24,46,47] leads us to speculate that AD is associated with an inability of neurons to degrade specific proteins or accumulated protein aggregates. The following outlines how such a defect in the UPS could occur.

A ubiquitin-conjugating enzyme, E2-25K/HIP-2, was reported to be a mediator of Aβ-mediated toxicity and proteasome inhibition in an animal model containing APP mutations [48]. A ubiquitin mutant, UBB^+1^ (a potent proteasome inhibitor observed in AD brains), was also found to functionally interact with E2-25K/HIP-2-mediated neurotoxicity [48]. In *in vitro* experiments, it was observed that Aβ could bind and inhibit the proteasome, thus blocking degradation of ubiquitin-conjugated proteins [49,50]. Furthermore, intraneuronally accumulated Aβ peptide in an APP/PSEN1 mutant neuronal cell culture was also shown to inhibit both the proteasome and deubiquitylating enzymes (DUBs) [51]. Ubiquitin immunoreactivity detected in axonal spheroids and dendritic compartments is associated with the elevated intraneuronal Aβ peptide and axonopathy observed in a transgenic mouse model of mutant APP/PSEN1 [52,53]. Results emerging from these studies support the idea that Aβ peptide's toxicity could be due to interference with ubiquitin conjugation or direct interaction with the proteasome itself.

It is also proposed that other causative factors in AD, such as paired helical filaments (PHFs) of tau protein, impair proteasome function [31]. In the brains of AD patients, proteasome function was shown to be reduced mostly in areas crucial for long-term memory formation such as the hippocampus, parahippocampal gyrus superior, middle temporal gyri and inferior parietal lobule, but not in other areas such as the occipital lobe [54]. Another study showed that PHFs of tau in brains of AD patients, as well as *in vitro* assembly of PHFs using human recombinant tau protein, both inhibited proteasome activity [55].

Taken together, evidence from the studies on early cognitive impairment in AD patients and animal models of AD, and the investigations on impairment of the UPS in AD, support a role for impairment of proteolysis in early synaptic dysfunction. Furthermore, there is considerable evidence supporting a role for the UPS in synaptic plasticity [2]. Although a secondary role for the UPS in AD pathology cannot be ruled out, given the role of the UPS in synaptic plasticity and in maintaining the integrity of the synapse, perturbations in these functions could be a primary cause of synaptic dysfunction and eventual synaptic loss in this disease.

The presence of ubiquitin and its association with tau in NFTs and senile plaques is a common factor in cases of AD [1]. Accumulated ubiquitin is also present in Lewy bodies, characteristic to some forms of the disease [26]. In all these cases, the role of tau and other putative target proteins in the pathogenesis of the disease is not understood. Also, it is still not clear whether aberration in proteolysis plays a causative role or only a secondary role, as described above [56]. Conflicting reports also exist on the E3 that ubiquitylates tau. Two reports claim that the ubiquitin ligase CHIP (carboxyl terminus of Hsp 70-interacting protein) targets the protein for degradation [57,58], whereas another study argues that the ubiquitin ligase TRAF6 is involved [59]. The discrepancy in the results could perhaps be resolved if the phosphorylation state of tau and the type of ubiquitin linkage that occurs with each ligase was known. CHIP has been shown to target hyperphosphorylated tau and TRAF6 to polyubiquitylate tau via Lys63 of ubiquitin (normally, polyubiquitylation occurs through Lys48 linkage). Thus, the two ligases could act on the same substrate under different conditions.

A more direct relationship between the ubiquitin system and pathogenesis of AD was established with the discovery of a frameshift mutation in the ubiquitin transcript, which results in ubiquitin with 20 extra amino acid residues at its C-terminus (UBB^+1^). The UBB^+1^ protein has been observed in the brains of AD patients [60]. UBB^+1^ is an efficient substrate for polyubiquitylation; however, it forms polyubiquitin chains that cannot be disassembled by DUBs. Polyubiquitin chains with UBB^+1^ potently inhibit the 26S proteasomal degradation of a substrate to which they are attached [61]. This report also suggests that the inhibitory activity of UBB^+1^ could be an important determinant of neurotoxicity and that it contributes to an environment that favors the accumulation of misfolded proteins [62,63].

In addition, other proteins implicated in AD, such as presenilins (PSENs) 1 and 2 (which are crucial for processing APP), are linked to the UPS. Specifically, both PSEN1 and PSEN2 are targets for UPS-mediated degradation [64]. It is not known, however, whether aberration in the degradation of PSEN1 and PSEN2 plays any role in the pathogenesis of AD.

## Disease targets and ligands

Development of therapeutic approaches and drugs that target components of the UPS would require a better understanding of the role of proteolysis in AD progression. There are many potential drug targets in the UPS, such as ubiquitin-conjugating enzymes, DUBs and the proteasome. Among the enzymes that conjugate ubiquitin to substrates, E3s are the best potential therapeutic targets because they mainly determine substrate specificity. Since the substrate binding region imparts specificity to E3s, allosteric modification of this region by small molecules, so that specific E3s have either increased or decreased affinity towards specific substrates, is one way of controlling accumulation of the ubiquitylated substrate [33]. Selective engineering of UPS components that enables modification of their delivery to a specific affected region and subsequent degradation of specific accumulated ubiquitylated proteins or protein aggregates, could provide an alternative approach to small allosteric molecules [33]. However, to date, no strong E3 candidate has been implicated in AD.

DUBs could also be potential drug targets if a definite role for these enzymes in AD is established. Modulation of specific DUBs by small molecules to enhance the deubiquitylation of polyubiquitin chains of mutant UBB^+1^ could be another possibility as these chains inhibit proteasomes in the AD brain [61]. A potential alternative strategy is to exploit the role of DUBs in synaptic plasticity to counteract the adverse effects on this process in the AD brain. For example, UCHL1 administration was utilized to rescue synaptic function [39] based on previous studies on the role of this protein in synaptic plasticity [38].

Another unexplored area for drug discovery is activation of the proteasome. Although many inhibitors of the proteasome are available, no effective drugs exist that can enhance the function of the proteasome. Since abnormal protein aggregation and proteasome inhibition is a common feature of AD and other neurodegenerative diseases, enhancement of proteasome activity by small molecules could be an efficient way to remove the aggregates that accumulate in the brain [33]. Removal of protein aggregates by the proteasome could be achieved by employing the following strategies: (1) by increasing the activity of the proteasome through up-regulating assembly of the 19S and 20S complexes; (2) by stimulating the recognition of ubiquitylated proteins in protein aggregates; (3) by overexpressing or modulating the chaperone activity of either chaperonins or ATPase subunits of 19S to unfold the aggregated proteins and (4) by stimulating the catalytic activity of the 20S core of the proteasome using small molecules [33]. Supporting this idea, a study showed that resveratrol, a naturally occurring polyphenol found in grapes and red wine, lowers the levels of Aβ in cell lines by promoting its proteasome-dependent degradation. This suggests a possible use for this compound in therapeutic intervention in AD [65].

With respect to ligands that modulate UPS components, there has been little progress. Even though proteasome inhibitors are being tested in clinical trial for cancer therapy [66], such compounds cannot be used in the nervous system because the role of the UPS in this system is more complex than in cancer [2,67]. As such, non-selective proteasome inhibition as a therapeutic strategy for neurodegenerative diseases could have many undesired side effects.

## New frontiers in drug discovery

Although several studies suggest a role for the UPS in AD, systematic and thorough studies are necessary to pinpoint how each component of the UPS could be linked to synaptic dysfunction or neurodegeneration in AD. Genetic evidence indicates that disruption of ubiquitin-mediated processes can lead to neurodegeneration; however, the relationship between the UPS and idiopathic neurodegenerative disorders is less clear. Basic research on the role of the UPS in normal brain function is likely to guide investigations into the links between UPS components and AD, which will allow identification of potential drug targets. Also, studies on genetic polymorphisms within UPS components, combined with functional validation in animal models, are likely to aid our understanding of how impairment of proteolysis contributes to pathogenesis of AD. This in turn will aid the development of therapeutic strategies. For example, even though polymorphisms in the intron of *SEL1L*[68], which encodes a component of the endoplasmic reticulum stress degradation pathway, have been found in Italian sporadic AD patients, it is not clear whether such polymorphisms have any effect on proteolysis. Similarly, it remains to be determined whether upregulation in AD brains of dactylidin, a putative RING finger ubiquitin ligase, has any functional consequences [69]. Since ubiquitin-like proteins play a role in the formation and regulation of autophagosomes [70], modulation of this autophagic-lysosomal pathway by UPS components could be another potential therapeutic strategy for removal of abnormal aggregated proteins seen in AD. A key area that requires further research is the development of small molecules to modulate the function of UPS components.

The role of the UPS in neurodegenerative diseases, including its connection to AD, is an intensive area of research worldwide. Proteolysis by the UPS is essential to normal cellular function and survival. Thus, it will be important to distinguish the roles of UPS components in specific neuronal functions and abnormalities such that a fuller understanding of UPS malfunction in diseases like AD can be obtained and therapeutic interventions be designed.

## Competing interests

The authors declare that they have no competing interests.

## Publication history

Republished from Current BioData's Targeted Proteins database (TPdb; ).
